# Supplementary material for: Implementing a Rural Natural Experiment: A Protocol for Evaluating the Impacts of Food Coops on Food Consumption, Resident’s Health and Community Vitality
Source: Methods Protoc. 2022 Apr 14;5(2):33. doi: 10.3390/mps5020033 (PMC9025453; doi:10.3390/mps5020033)
Supplement: Supplementary file 1 [file mps-05-00033-s001.zip › mps-1595146 Document S4-Interview guide.pdf]

Semi-structured interview guide for key informants

<table>
<tr><th colspan="2">Interview questions, depending on the key informant and the community</th></tr>
<tr><td colspan="2"><h2>1-Informant's role</h2>- What is your connection to the coop/project?</td></tr>
<tr><td>Citizen member of the board of directors or provisional committee :
- What is their role in the creation and development of the cooperative project</td><td>Other types of informants :
- What is their role in the project?</td></tr>
<tr><td colspan="2">- How long have you been involved?
- Why did you get involved in the project?</td></tr>
<tr><td colspan="2"><h2>2-Description of the project</h2>- What does the project consist of?
    o Other goods and services offered to the co-op (were they an integral part of the project from the beginning?)
- What need(s) are being met? How were these needs identified?
    o Food accessibility
- Food offer at the coop?
    o Convenience store style, small grocery store, etc?</td></tr>
<tr><td>Citizen member of the board of directors or provisional committee :
- Why is the cooperative project important to the community?
- Why did you choose the cooperative model?
- Food offer at the coop :
    o Types of food available?
    o How were the foods sold selected?
    o Local food?
- Number of members and/or % of residents of the municipality who are members of the coop?
- Choice of location, land?
- Employees? Volunteers involved?</td><td>Other types of informants :
- Why is this project important to the community? And for the municipality?</td></tr>
<tr><td colspan="2"><h2>3-Project creation process</h2>- Please describe the process of creating the project.</td></tr>
</table>

Citizen member of the board of directors or provisional committee :

- **Can you tell us what the major steps were** (survey of the population, formation of a provisional committee, feasibility study, business plan, financing, formation of the board, etc.)?
- **Did you have access to a guide describing the various steps to be taken to guide you in the process of setting up the cooperative** (information and methods)? If so, **which one?** Ask about the relevance of having one.
- Need for a project manager (government or non-profit funded)?
- Did you have the information you needed, when you needed it?

Other types of informants :

- **What were the major steps and what were you involved in?**
- **Discuss the administrative process.**

4-Community mobilization

- **Can you tell me about the mobilization of citizens around the project ?**
- What is the c~~C~~ontext of the project's creation
- How did the initiative start? Who initiated the project?
- Were the needs raised by the community?
  - Community survey
- Role of the population in the creation process?

5-Collaboration

- **Partnerships**
  - **Are there any key players or organizations that are also associated with this project?**
- Is the city or the- Regional County Municipality involved in any way in the project?
- How did you work together on the project?
- Did the project create new relationships~~links~~ between the actors?
- Are there any partnerships with other businesses around?
- **Have the public health actors been involved or solicited?**
- Did the local production and distribution actors collaborate in the project?
- Are there other actors who did not collaborate? And how do you think their contribution could have benefited the project? How could they be brought together?

Citizen member of the board of directors or provisional committee :

- How were the links created between you and the municipality?
- ~~E~~Any external support?

Other types of informants :

- How were the links created with the instigators of the project?

6-Facilitating factors

- **In your opinion, what are the most important elements that explain why the cooperative's project is progressing (or has progressed well)?**
  - *Political climate, role of the media, external support, social networks, leadership, complexity, adaptation, knowledge, resistance to change, strategy, capacity and skills, funding, shared vision ?*
- **For the steps already taken, what elements could have helped you develop your project more easily, more quickly?**

7-Barriers

- **What would you say are the main barriers or obstacles to your project? How have you been able to overcome them so far?**
- In your opinion, what means could/should be put in place to limit the barriers and to better support coop projects?

8-Anticipated impacts of the implementation of the coop on the community

- How can such a project benefit its community?
- What are the impacts of the coop on the food supply of the citizens? On the well-being of the citizens? On the vitality of the community? On the economy of the region? On the population in general?
- What is the more general role of the coop in the community?

9-The project in the context of COVID-19

- In your opinion, what are the impacts of COVID on the coop's project (mobilization, motivation, deadlines, etc.)? (In the case of an open coop: functioning, role in the resilience of the population)
-

10-Sustainability of the project

- In your opinion, how do you ensure or will you ensure the sustainability of the coop ?
- -What mechanisms and resources have been put in place (recruitment of new leaders, ongoing training of members, partnerships, etc.)?

11-Other

- Are there any other issues related to the coop project that you would like to raise? (relocation, renovation, collaboration, solicitation of actors, etc.)
- **Do you have other elements in mind that are related to the coop that we have not discussed?**
- Do you have any advice to give to a group that wants to set up a cooperative in their community?
- **Do you know of any actors who could provide their point of view on the process of a cooperative project?**
