# Supplementary material for: Implementing a Rural Natural Experiment: A Protocol for Evaluating the Impacts of Food Coops on Food Consumption, Resident’s Health and Community Vitality
Source: Methods Protoc. 2022 Apr 14;5(2):33. doi: 10.3390/mps5020033 (PMC9025453; doi:10.3390/mps5020033)
Supplement: Supplementary file 1 [file mps-05-00033-s001.zip › mps-1595146 Document S2-Consent form.pdf]

Participant Consent Form

Who is leading this project?

Éric Robitaille, Ph.D., Specialized Scientific Advisor and Assistant Clinical Professor School of Public Health, Department of Social and Preventive Medicine, University of Montreal, INSPQ, 190 Crémazie Blvd. East, Montreal, Quebec, H2E1P2, tel: 514-864-1600 ext. 3561.

Describe this project

We invite you to participate in a research project on the effects of the implementation of a food cooperative on nutrition and health and community vitality (EffICAS Study). However, before agreeing to participate in this research project, please take the time to read, understand and carefully consider the following information. the following information.

The main objective of this project is to analyze the effects of the implementation of a food cooperative in a rural community and in an area with low access to healthy food, food quality, health and quality, health and community vitality.

If I participate, what do I have to do?

As part of this project, you will be asked to complete an online questionnaire twice. Completing the questionnaire should take about 30-40 minutes each time.

Are there any risks or benefits to participating in this research?

By participating, you will have the opportunity to contribute to the advancement of knowledge about the impact of food retailing on health and community health and community vitality. You will receive a summary report of the research findings. There will be no personal inconvenience as a result of your participation in this study. There are no known risks to participating in this research. You are free to participate. A compensation of $25 in the form of a gift card will be offered for each completed questionnaire. It will be sent to you by email.

Will my personal information be protected?

The information collected during this research will be treated as strictly confidential. No results will contain your name or personal information and no one personal information and no one will be able to identify you. A system will be used for this purpose. Your name and any other identifying information (for example, your address) that could identify you will not be mentioned. The data from the questionnaire will be stored on a server at the server of the Institut national de santé publique du Québec (INSPQ) and protected by a password: only the principal investigator, the co-investigators and the research assistants will have access to it. All research material will be destroyed seven years after the end of the study, according to the procedures. The Institut national de santé publique du Québec will keep, for other research projects. The results will be used exclusively by the researchers in scientific communications or teaching and by the Institut national de santé publique du Québec and the Ministère de la and the Ministère de la Santé et des Services sociaux du Québec, which are funding this research. Although some information may identify you, the information collected during this research will be treated as strictly confidential.

Do I have to answer all the questions and complete the research to the end?

Your participation in this project is voluntary. You have the right to withdraw from the project at any time, without consequence. Your withdrawal from the study will have no impact on you or your situation. But to financial compensation, all questions must be answered.

Who can I talk to if I have questions during the study?

If you have any questions, you can contact Éric Robitaille, principal investigator researcher, at the following number: 514-864-1600 - extension 3561, or at the following address info.efficas@inspq.qc.ca

This project has been approved by the University of Montreal's and Health Research Ethics Committee. If you have any concerns about your rights or the responsibilities of the researchers regarding your participation in this project, you can contact the committee by e-mail at cerses@umontreal.ca or visit the website: http://recherche.umontreal.ca/participant.

If you have any complaints regarding your participation in this research you may contact the Ombudsman of the Université de Montréal at telephone number 514-343-2100 or at the following e-mail address ombudsman@umontreal.ca. The ombudsman accepts collect calls.

How do I give my consent to participate in the study?

All you have to do is:

- Read the participant statement;
- Enter your email address;
- Enter your full name;
- Check "yes" after "I agree to participate in the study";

A copy of the consent form will be sent to you by email for your reference.

CONSENT

Participant Declaration

I understand that I can take my time to think about whether or not I want to participate.

- I can ask questions of the research team and demand satisfactory answers.
- I understand that by participating in this research project, I am not waiving any of my rights and that this does not relieve the researchers of their responsibilities.
- I have read this information and consent form and agree to participate in the research project.

E-mail: ________________________________

Full name: ________________________________

I agree: Yes No

This project has been approved by the Health Research Ethics Committee of the Université de Montréal.

Montreal Health Research Ethics Board.

Project no CERSES-20-041-D
