# Supplementary material for: Implementing a Rural Natural Experiment: A Protocol for Evaluating the Impacts of Food Coops on Food Consumption, Resident’s Health and Community Vitality
Source: Methods Protoc. 2022 Apr 14;5(2):33. doi: 10.3390/mps5020033 (PMC9025453; doi:10.3390/mps5020033)
Supplement: Supplementary file 1 [file mps-05-00033-s001.zip › mps-1595146 Document S3-Questionnaire.pdf]

COOP Study Questionnaire

Welcome to the COOP Survey!

You are about to fill out a map questionnaire that asks you where you usually go to do certain activities, such as grocery shopping.

For each activity, we will first ask you to indicate on a map the location(s) where you do these activities. Then we will ask you to provide information about those locations.

This questionnaire will take between 30 and 40 minutes to complete.

We ask that you take the time to read each question carefully and give the answer that best reflects your opinion/current situation. There is no right or wrong answer. We really want to hear your personal perspective.

<u>You may exit the survey at any time and resume where you left off (your answers will not be lost), using the same email address and password that was provided to you by email.</u>

Instructions

An answer must be entered for each question asked. If you do not answer a question on a page, you will not be able to move to the next page and an error message indicating that the field is not filled in will appear when you click on the "next" button.

You can navigate through the questionnaire by clicking on the "previous" and "next" buttons at the bottom of the screen. The appearance of the "next" button changes from white to orange when all fields on the page are completed.

Questions that ask you to identify a date allow you to select it using a calendar. You can also enter the year and month directly, in that order (e.g. 2011-06), for a quicker response.

If you are unable to complete the entire survey in one sitting, you can click the "Save for Later" button to save your answers and log back into the survey using the username and password that was provided to you by email.

How to Use the Map

In this questionnaire, you will use a map like this one to confirm your location, to locate and identify places where you do activities, and to plot areas or routes. To familiarize yourself with the map tools, please follow these instructions.

To search for or add a place:

You can enter the name of the location in the search bar at the top left of the map. One or more symbols 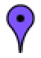 will appear on the map, depending on the locations associated with the keywords you used. Then simply click on the symbol in the correct location to select it.

You can use the zoom buttons below the search bar to move around the map more easily and locate the location you are looking for.

To add an unlisted location (for example, your home), you can click on the symbol 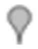 .

Once the location is confirmed, the marker will turn red like this: 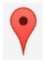

Note that the door number displayed on a confirmed location (e.g., your home) may be slightly different from what you entered. This is a normal inaccuracy generated by the mapping process. If the difference between reality and the map is too great, you can use the gray marker to identify the exact location of the place you want to identify.

Now it's your turn! Practice by looking for the Dominique-Savio School or another location of your choice.

1.YOUR HOME AND NEIGHBORHOOD

Q1. So let's start with your residence. What is your address?

(Your primary place of residence, where you live today.)

Number (and suffix, if any) (e.g., 302, 151 B, 16 1/2)
Street name, type of street (e.g., AV = Avenue), direction (e.g., N = North)
Apartment/unit
City, town, village, aboriginal reserve
Postal code

A map appears with a house icon at the address entered by the participant.

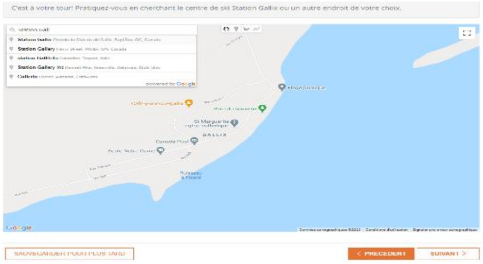

Q2. Does this location correspond to your home?

Yes
No

If so, select "confirm" and then "next". Otherwise, you can move the marker to your home address. Once the location identified is your home address, select "next".

Q3. How long have you lived at this address?
Use the calendar below to select the date (YYYY-MM)

2. SHOPPING ACTIVITIES/Main STORE

Q4. Currently, when you make your largest food purchases for the home, where do you primarily go? This is your primary retailer. Please indicate this on the card.

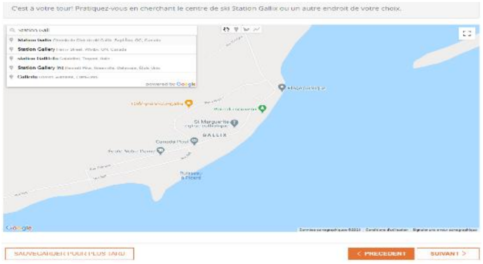

Q4_PC Please answer the following questions about your primary retailer.

A Name of the location :

B How do you usually go there? (More than one answer possible)

By car and you drive
By car and someone else drives
By cab
By foot
By bicycle
By public transport
Other
If other, please specify:

Q5. What is the MAIN reason you shop at this location?

The quality of the products (fresh fruits and vegetables, meats)
The choice of products offered
The low prices, discounts and promotions offered
To buy in bulk or in bulk
The services offered by the store (telephone orders, good customer service, delivery service, etc.)

3. SHOPPING ACTIVITIES/Secondary Supply

Section Intro: The following questions ask about where you buy your food, <u>other than from your primary retailer</u>.
For example, grocery store, farmers market, bakery, specialty store, convenience store.

Q6. do you go to the grocery store or supermarket at least once a month?
Indicate only places other than your primary retailer.

Yes - Go to question 12
No - Go to question 13

Q7. Can you locate the grocery store(s) or supermarket(s) where you go at least once a month?
You may add up to 5 locations.

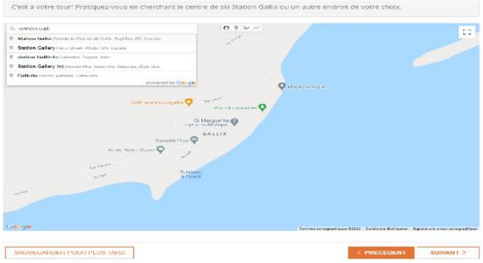

Q12_PC Please answer the following question for each grocery store.

A Name of the location :

B How do you usually go there? (More than one answer possible)

By car and you drive
By car and someone else drives
By cab
By foot
By bicycle
By public transport
Other
If other, please specify:

Q8. In season, do you go to a farmers' market at least once a month?

Yes - Go to question 14
No - Go to question 15

Q9. Can you locate this (these) public market(s)?
You may add up to 5 locations.

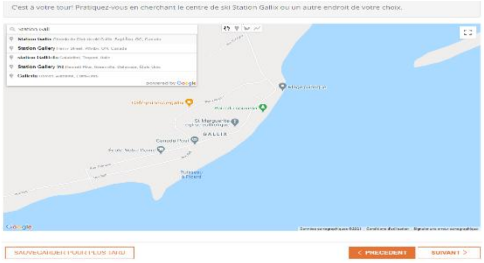

Q14_PC Please answer the following question for each public market.

A Name of the location :
B How do you usually go there? (More than one answer possible)

By car and you drive
By car and someone else drives
By cab
By foot
By bicycle
By public transport
Other
If other, please specify:

Q10. Do you go to a specialty food store at least once a month?
For example, a fruit store, a fish store, a butcher shop (e.g. Boucherie Mar-Gil Ltée) or a bakery.

Yes - Go to question 16
No - Go to question 17

Q11. Can you locate this (these) specialty food store(s)?
You may add up to 5 locations.

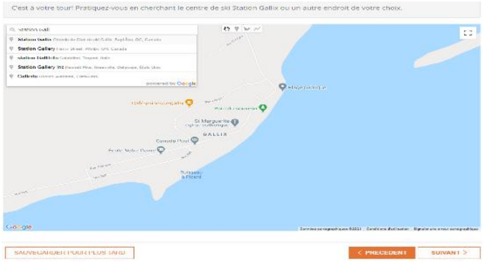

Q16_PC Please answer the following question for each specialty food store.

A Name of the location :

B How do you usually go there? (More than one answer possible)

By car and you drive
By car and someone else drives
By cab
By foot
By bicycle
By public transport
Other
If other, please specify:

Q12. Do you go to a convenience food store at least once a month?

Yes - Go to question 18
No - Go to question 19

Q13. Can you locate this (these) convenience store(s)?

You may add up to 5 locations.

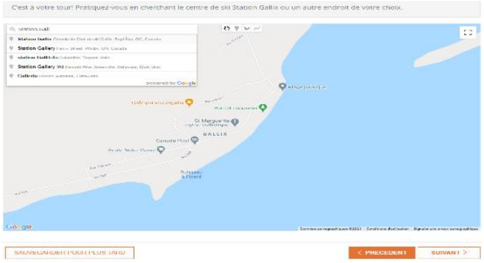

Q18_PC Please answer the following question for each convenience store.

A Name of the location :

B How do you usually go there? (More than one answer possible)

By car and you drive
By car and someone else drives
By cab
By foot
By bicycle
By public transport
Other
If other, please specify:

Q15. Do you go at least once a month to eat or drink at a restaurant, café or snack bar
Yes - Go to question 20
No - Go to question 21

Q16. Can you locate this (these) café or snack bar?
You may add up to 5 locations.

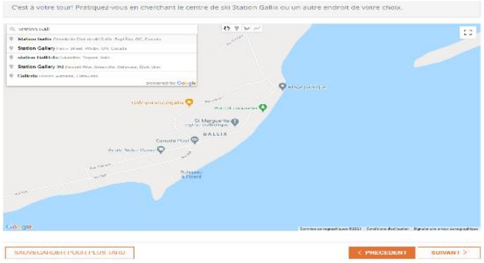

Q20_PC Please answer the following question for each place identified.

A Name of the location :
B How do you usually go there? (More than one answer possible)
By car and you drive
By car and someone else drives
By cab
By foot
By bicycle
By public transport
Other
If other, please specify:

Q17. Do you go at least once a month get take out at a restaurant?
Yes - Go to question 22
No - Go to question 23

Q18. Can you locate this (these) restaurant(s)?
You may add up to 5 locations.

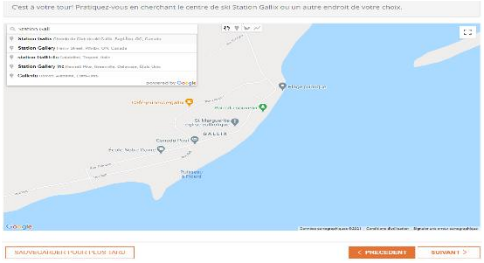

Q22_PC Please answer the following question for each restaurant.

A Name of the location :
B How do you usually go there? (More than one answer possible)
By car and you drive

By car and someone else drives
By cab
By foot
By bicycle
By public transport
Other
If other, please specify:

4. SHOPPING ACTIVITIES/Alternative Supply

Q19. During the summer of 2020, did you ever purchase fruits or vegetables directly from a producer (roadside stand, U-pick, basket service)?

Yes - Go to question 24
No - Go to question 25

Q20. Where is the kiosk, U-pick or drop-off location for the fruit and vegetable basket service?

You may add up to 5 locations.

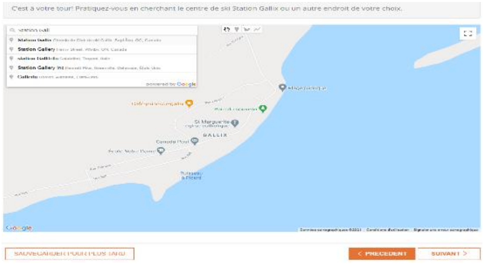

Q24_PC Please answer the following question for each service you have identified.

A Name of the location :
B How do you usually go there? (More than one answer possible)
By car and you drive
By car and someone else drives
By cab
By foot
By bicycle
By public transport
Other
If other, please specify:

Q21. During the summer of 2020, did you grow vegetables, herbs, or fruit at home or in a community garden?

Yes, at home - go to question 27
Yes, in a community garden - go to question 26
No - go to question 27

Q22. Where is this community garden?

You may add up to 5 locations.

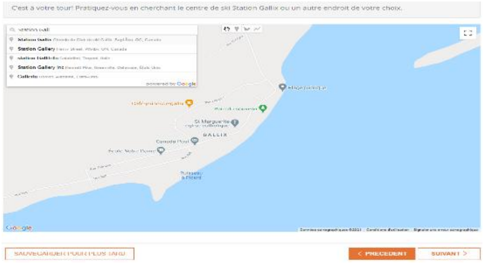

Q26_PC Please answer the following question for each community garden.

A Name of the location :

B How do you usually go there? (More than one answer possible)

- By car and you drive
- By car and someone else drives
- By cab
- By foot
- By bicycle
- By public transport
- Other
- If other, please specify:

Q23. In the past 12 months, have you (or anyone in your household) visited or used a food pantry (e.g., food bank, soup kitchen) to get food?

- Yes - Go to question 28
- No - Go to question 29

Q24. Where is this food pantry?

You may add up to 5 locations.

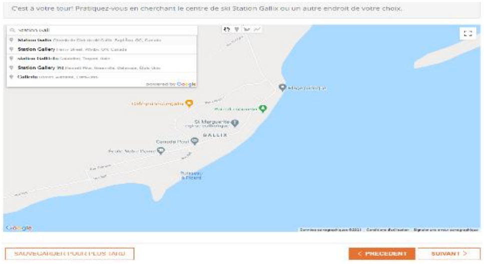

Q27_PC Please answer the following question for each service you have identified.

A Name of the location :

B In the past 12 months, on average, how often did you go to this food pantry?

- Weekly - Go to Q27_PC-C
- Every month, but not every week - Go to Q27_PC-C
- A few times a year, but not every month - Go to Q27_PC-C

I don't know- Go to Q27_PC-D

C How often?

D How do you usually go there? (More than one answer possible)

By car and you drive

By car and someone else drives

By cab

By foot

By bicycle

By public transport

Other

If other, please specify:

5. Perception of the food environment

Section introduction: *This section is designed to get your opinion on the accessibility and availability of healthy foods in your municipality. Healthy foods are those listed in Canada's Food Guide, i.e., vegetables and fruit, whole grains (bread, cereals), protein foods (meat, milk, yogurt, legumes) and water.*

You must check the box that best corresponds to your choice. Some questions may seem repetitive. However, it is important to answer all the questions.

Q25. Please indicate your level of agreement or disagreement with the following statements

(Strongly disagree/ Strongly disagree/ Neither disagree nor agree/ Somewhat agree/ Agree)

A I consider the amount of healthy food offered by my main retailer to be sufficient.

B I consider the variety of healthy foods offered by my main retailer to be sufficient.

C I consider the quality of the healthy foods offered by my main retailer to be acceptable.

D I consider the cost of the healthy foods offered by my main retailer to be affordable.

E I consider that I have easy access to a food retailer with a wide variety of foods, close to my home.

F I feel that the media's information on food has a positive influence on my diet.

G I consider that fast food restaurants are easily accessible near my home.

H I consider that fast food restaurants are easily accessible near my workplace.

I I consider that I have easy access to junk food or snacks in my workplace (chips, chocolate, pastries).

6. Fruit and vegetable consumption

Section Intro: The following questions are about the fruits and vegetables you have eaten or drank in the past month. Think about everything you ate as a meal or snack, both at home and away.

Q26. In the past month, have you drunk 100% PURE fruit juices, such as pure orange juice, apple juice, or pure juice mixes? Please exclude fruit-flavored drinks with added sugar or other sweeteners or fruit punches.

Yes, every day - Go to question 30_P
Yes, every week - Go to question 30_P
Yes, every month - Go to question 30_P
Yes, less than once a month - Go to question 31
No - Go to question 31
Prefer not to answer / Don't know - Go to question 31

Q30_P How many times?

Q27. In the past month, not including juice, did you eat any fruit?
Please include frozen, dried or canned fruit.

Yes, every day - Go to question 31_P
Yes, every week - Go to question 31_P
Yes, every month - Go to question 31_P
Yes, less than once a month - Go to question 32
No - Go to question 32
Prefer not to answer / Don't know - Go to question 32

Q31_P How many times?

Q28. In the past month, have you eaten dark green vegetables such as broccoli, green beans, peas, and green peppers or leafy greens, including romaine lettuce or spinach? Please include frozen or canned vegetables and cooked vegetables in soups or mixed into salads.

Yes, every day - Go to question 32_P
Yes, every week - Go to question 32_P
Yes, every month - Go to question 32_P
Yes, less than once a month - Go to question 33
No - Go to question 33
Prefer not to answer / Don't know - Go to question 33

Q32_P How many times?

Q29. In the past month, have you eaten orange vegetables such as carrots, orange peppers, sweet potatoes, pumpkin or squash? Please include frozen or canned vegetables and vegetables cooked in soups or mixed into salads.

Yes, every day - Go to question 33_P
Yes, every week - Go to question 33_P
Yes, every month - Go to question 33_P

Yes, less than once a month - Go to question 34
No - Go to question 34
Prefer not to answer / Don't know - Go to question 34

Q33_P How many times?

Q30. In the past month, have you eaten non-fried potatoes?
Yes, every day - Go to question 34_P
Yes, every week - Go to question 34_P
Yes, every month - Go to question 34_P
Yes, less than once a month - Go to question 35
No - Go to question 35
Prefer not to answer / Don't know - Go to question 35

Q34_P How many times?

Q31. In the past month, excluding the greens and orange vegetables and potatoes you mentioned earlier, have you eaten ANY OTHER types of vegetables? Examples include cucumbers, celery, corn, cabbage and vegetable juices.
Yes, every day - Go to question 35_P
Yes, every week - Go to question 35_P
Yes, every month - Go to question 35_P
Yes, less than once a month - Go to question 36
No - Go to question 36
Prefer not to answer / Don't know - Go to question 36

Q34_P How many times?

7. Food quality

Section Intro: For the following questions, we ask you to answer yes or no.
Please refer to your usual consumption.

Q32. Do you eat deli meats (e.g. sausages, cold cuts, terrines) more than twice a week?
Yes Go to question Q37
No Go to question Q44

Q33. Do you eat more than one apple a day?
Yes Go to question Q42
No Go to question Q38

Q34. Do you eat salad 5 or more times a week?
Yes Go to question Q41
No Go to question Q39

Q35. Do you eat nuts 2 or more times a week?
Yes Go to question Q40
No Go to block 8

Q36. Do you drink coffee or tea more than twice a day?

Yes Go to block 8
No Go to block 8

Q37. Do you consume humus three or more times a month?
Yes Go to Block 8
No Go to block 8

Q38. Do you eat peanut butter at least once a month?
Yes Go to question 43
No Go to Block 8

Q39. Do you drink fruit juice most days?
Yes Go to Block 8
No Go to block 8

Q40. Do you consume soft drinks more than once a month (1 can = 1 serving)?
Yes Go to question 48
No Go to question 45

Q41. Do you drink a glass of 2% milk every day?
If you drink skim, 1% or 3.25% milk, please check no.
Yes Go to block 8
No Go to question 46

Q42. Do you eat more than one apple per week?
Yes Go to block 8
No Go to question 47

Q43. Do you eat onions more than once a week?
Yes Go to block 8
No Go to block 8

Q44. Do you eat nuts 3 or more times a month?
Yes Go to question 49
No Go to question 53

Q45. Do you drink fruit juice 5 or more days a week?
Yes Go to question 52
No Go to question 50

Q46. Do you eat French fries more than once a week?
Yes Go to question 51
No Go to block 8

Q47. Do you eat fish 3 or more times a week?
Yes Go to block 8
No Go to block 8

Q48. Do you eat humus 2 or more times a month?
Yes Go to block 8
No Go to block 8

Q49. Do you eat whole grain bread most days?
Yes Go to question 54
No Go to block 8

Q50. Do you eat pasta every other day or more often?
Yes Go to block 8
No Go to question 55

Q51. Do you eat broccoli more than once a week?
Yes
No

8. Food security

Section Intro: The next questions deal with your household's food situation in the last 12 months.

Q52. Indicate whether the following statements are SOMETIMES true, SOMETIMES true, or NEVER true for you and other members of your household in the past 12 months.

A You were afraid you would run out of food before the next cash flow.

B All the food you bought was eaten and there was no money to buy more.

C You could not afford to eat balanced meals.

If (Q56A, Q56B or Q56C = 1 or 2)], go to question Q57

If (Q56A, Q56B AND Q56C = 3 or 99)], go to question Q62

Q53. In the past 12 months, have you or any other adult in your household ever cut back on your portion size or skipped meals because there was not enough money for food?

Yes - Go to question Q57_P
No - Go to question Q58
Don't know / Refused - Go to question Q58

Q57_P How often did this happen?

Almost every month
Some months but not every month
Only 1 or 2 months
Don't know / Refused

Q54. In the past 12 months, have you (yourself) ever eaten less than you think you should have because there was not enough money to buy food?

Yes
No
Don't know / Refused

Q55. In the past 12 months, have you (yourself) ever been hungry and couldn't eat because you couldn't afford enough food?

Yes
No
Don't know / Refused

Q56. In the past 12 months, have you (yourself) lost weight because you couldn't afford food?

Yes
No
Don't know / Refused

If (Q57, Q58, Q59 or Q60 = 1), go to question Q61
If (Q57, Q58, Q59 and Q60 = 2 or 99), go to question 62

Q57. In the past 12 months, have you or other adults in your household gone a whole day without eating because there was not enough money for food?

Yes - Go to question Q61_P
No - Go to question Q62
Don't know / Refused - Go to question Q62

Q61_P How often did this happen?

Almost every month
Some months but not every month
Only 1 or 2 months
Don't know / Refused

9. Community vitality and well-being

Section Intro1: The next questions are about living conditions and community life in *Rivière-Pentecôte*.

Section Intro2: Reflecting on your experiences in your community, indicate the extent to which you agree or disagree with each of the following statements?

Q75. Middle-income earners can afford to buy a home in this community.

Strongly agree
Somewhat agree
Neither agree nor disagree
Somewhat disagree
Strongly disagree
Don't know
Prefer not to answer

Q75. Young adults aged 25-34 consider Rivière-Pentecôte a desirable place to live.

Strongly agree
Somewhat agree
Neither agree nor disagree
Somewhat disagree
Strongly disagree
Don't know
Prefer not to answer

Q76. You see many active and healthy older people in Peninsula River.

Strongly agree
Somewhat agree
Neither agree nor disagree
Somewhat disagree

Strongly disagree
Don't know
Prefer not to answer

Q77. For newcomers to Rivière-Pentecôte and those who want to, there are interesting opportunities to get involved and make a difference.

Strongly agree
Somewhat agree
Neither agree nor disagree
Somewhat disagree
Strongly disagree
Don't know
Prefer not to answer

Q78. On issues that divide the community of Rivière-Pentecôte, there is civilized debate, good information flow and inclusive and respectful consultation.

Strongly agree
Somewhat agree
Neither agree nor disagree
Somewhat disagree
Strongly disagree
Don't know
Prefer not to answer

Q79. The community of Rivière-Pentecôte considers sustainable development in its community planning.

Strongly agree
Somewhat agree
Neither agree nor disagree
Somewhat disagree
Strongly disagree
Don't know
Prefer not to answer

Q80. Residents are proud of Rivière-Pentecôte. They always promote it to outsiders.

Totally agree
Somewhat agree
Neither agree nor disagree
Somewhat disagree
Strongly disagree
Don't know
Prefer not to answer

Q81. There is a vibrant meeting place or community core in Rivière-Pentecôte.

Strongly agree
Somewhat agree
Neither agree nor disagree
Somewhat disagree
Strongly disagree
Don't know
Prefer not to answer

Q82. Those with skills and training can find a well-paying job in or near Pentecostal River.
Strongly agree
Somewhat agree
Neither agree nor disagree
Somewhat disagree
Strongly disagree
Don't know
Prefer not to answer

Q83. People who live in Rivière-Pentecôte are likely to act on an important local need.

Strongly agree
Somewhat agree
Neither agree nor disagree
Somewhat disagree
Strongly disagree
Don't know
Prefer not to answer

Q84. I can influence decisions that affect the community of Rivière-Pentecôte.

Strongly agree
Somewhat agree
Neither agree nor disagree
Somewhat disagree
Strongly disagree
Don't know
Prefer not to answer

Q85. It would take a long time for Pentecost River to return to normal if something serious happened that affected everyone, such as a severe storm, flood, or violent crime.

Strongly agree
Somewhat agree
Neither agree nor disagree
Somewhat disagree
Strongly disagree
Don't know
Prefer not to answer

Q86. The people of Rivière-Pentecôte have an impact when they work together to help their community.

Strongly agree
Somewhat agree
Neither agree nor disagree
Somewhat disagree
Strongly disagree
Don't know
Prefer not to answer

Q87. When Pentecostal River faces a challenge, it strengthens the community spirit.

Strongly agree
Somewhat agree
Neither agree nor disagree
Somewhat disagree
Strongly disagree
Don't know
Prefer not to answer

Q88. Overall, how satisfied are you with the quality of life in Rivière-Pentecôte?

Strongly dissatisfied
Dissatisfied
Neither dissatisfied nor satisfied
Satisfied
Strongly satisfied

Q89. How much do you like living in Rivière-Pentecôte?

Not at all
Somewhat
Moderately
A lot

Q90. When you think about the living conditions in Rivière-Pentecôte, are the conditions...

Getting worse
Staying about the same
Getting better

Q91. In the future, do you think conditions in Riviere-Pentecost will be...

Worse than today
Somewhat less worse
Neither worse nor better
A little better
Better than today

Q92. How desirable do you consider Rivière-Pentecôte to be as a place to live?

Not very desirable/inviting
Somewhat desirable/inviting
Desirable/inviting
Very desirable/inviting
One of the best places to live in Quebec

10. Socio-demographic and economic data

Section introduction: The next questions deal with different socio-demographic characteristics.

Q58. What was your sex at birth?

Sex refers to the sex assigned at birth.

Male
Female
Prefer not to answer
Don't know

Q59. What gender do you identify with?

Gender refers to current gender, which may differ from the sex assigned at birth or the sex recorded in legal documents.

Male
Female
Other
Prefer not to answer
Don't know
If other, please specify:

Q60. In general, would you say your health is :

Excellent
Very good
Good
Fair
Poor
Don't know
Prefer not to answer

Q61. In general, do you experience the following physical difficulties:

Yes No Prefer not to answer / Don't know

Difficulty lifting and carrying heavy loads (e.g. groceries)

Difficulty walking and moving around in small spaces (e.g. grocery store)

Q62. How tall are you?

Q63. What is your weight?

Q64. Are you currently pregnant?

Yes
No
Don't know
Prefer not to answer / Not applicable

Q65. Were you born in Canada?

Yes - Go to question Q76
No - Go to question Q75
Don't know - Go to question Q75
Prefer not to answer - Go to question Q75

Q66. How many years have you lived in Canada?

Less than 5 years
5 to 10 years
More than 10 years
Don't know
Prefer not to answer

Q67. What language do you speak most often at home?

If you speak two languages equally often, check both choices.

- French
- English
- Other
Don't know
Prefer not to answer

Q68. Which type of situation best fits your current household composition?

Person living alone - Go to question 80
Couple without children at home - go to question 80
Couple with children at home (children of all ages) - Go to question 79
Single parent family (children of all ages) - Go to question 79
Family with other persons (related or not) - Go to question 78
Related persons only (brothers, sisters, etc.) - go to question 78
Non-relatives only (roommates) - go to question 78
Other (multiple families, etc.) - go to question 78
Don't know - go to question 78
Prefer not to answer - Go to question 78

Q69. Including yourself, how many people live in your household?

One
Two
Three
Four
Five
Six
Seven
Eight
Nine
Ten or more
Don't know
Prefer not to answer

Q70. How many children are there in your household?

None
One
Two
Three
Four
Five
Six
Seven
Eight
Nine
Ten or more
Don't know
Prefer not to answer

Q71. Do you or anyone in your household...?

Owning or paying for this dwelling
Renting, even if no cash rent is paid
Don't know
Prefer not to answer

Q72. What is the highest level of education you have completed?

If you have a diploma from another country, choose its equivalent in the Quebec school system.

Primary school or less
Some high school
High school diploma
Diploma or certificate from a trade or vocational school
Commercial college diploma

CEGEP diploma
University undergraduate degree (certificate, minor, major, bachelor)
Master's, doctorate or graduate degree
Other
Don't know
Prefer not to answer

Q73. During the past 12 months, what was your MAIN employment status?

Full-time worker
Part-time worker (less than 30 hours)
Student
Retired
Householder
On parental leave
Employment Insurance recipient
Social assistance recipient
On disability or sick leave
Other
Don't know
Prefer not to answer

Q74. Can you please indicate the approximate total income of all household members from all sources, before taxes and other deductions?

Remember to include all amounts such as Canada Child Benefits, investment income, etc.

Less than $10,000
$10,000 to less than $20,000
$20,000 to less than $30,000
$30,000 to less than $40,000
From $40,000 to less than $50,000
From $50,000 to less than $60,000
From $60,000 to less than $80,000
From $80,000 to less than $100,000
From $100,000 to less than $120,000
$120,000 to less than $140,000
$140,000 or more
Don't know
Prefer not to respond

FIN1 Thank you, you have completed the survey!
We would like to remind you that you may be asked again in a few months for a second survey.
Before you leave, please indicate which $25 gift card you would like to receive.
Pharmaprix (applicable in store or online)
La Cordée, outdoor equipment. (applicable in store or online.)

Leslibraires.ca, paper and digital books (applicable in store, at Côte-Nord bookstore in Sept-Îles, or online)

FIN2 Thank you for your participation!
A summary of the results of the research will be sent to you by email later this year.
