# Supplementary figures and images for: Implementing a Rural Natural Experiment: A Protocol for Evaluating the Impacts of Food Coops on Food Consumption, Resident’s Health and Community Vitality
Source: Methods Protoc. 2022 Apr 14;5(2):33. doi: 10.3390/mps5020033 (PMC9025453; doi:10.3390/mps5020033)

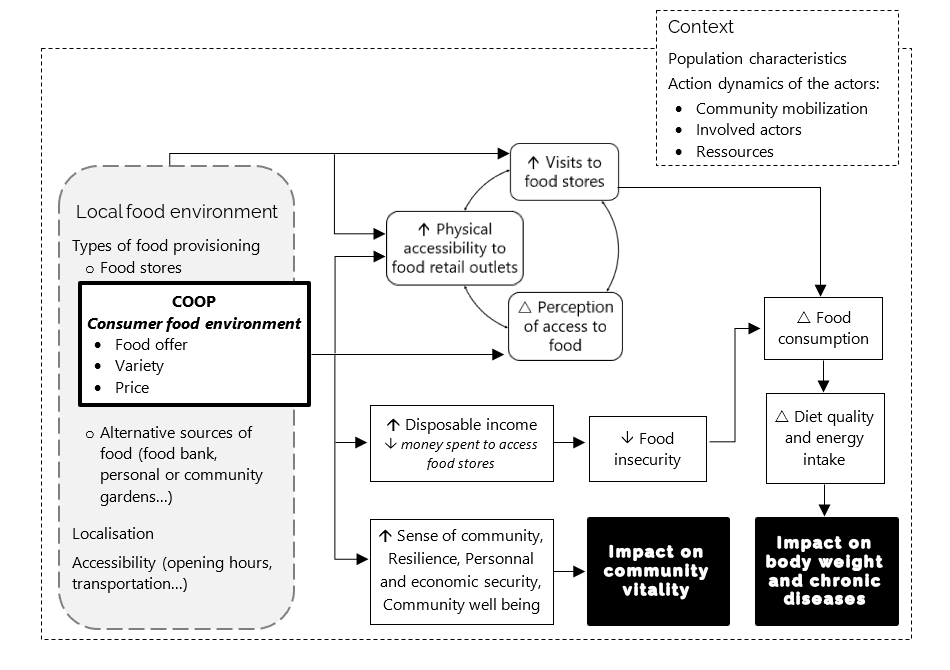

Supplement: Supplementary file 1 [file mps-05-00033-s001.zip › mps-1595146 Figure S1-Logic model.png]
